# COVID-19-Associated Autoimmune Disease: A Rare First Case Report of Acute Motor Axonal Neuropathy Variant of Guillain-Barre Syndrome in a Woman Patient in New York City

**DOI:** 10.7759/cureus.22290

**Published:** 2022-02-16

**Authors:** Zaheer Qureshi, Sameer Kandhi, Neeti Prasai, Faryal Altaf, Manjeet Dhallu

**Affiliations:** 1 Internal Medicine, Icahn School of Medicine at Mount Sinai, New York, USA; 2 Internal Medicine, BronxCare Health System, Bronx, USA; 3 Neurology, BronxCare Health System, Bronx, USA

**Keywords:** axonal neuropathy, autoimmune, guillain-barre, covid-19, coronavirus

## Abstract

Novel outbreaks with COVID-19 can cause multiple systemic manifestations, including autoimmune disease. Among all the infections, respiratory complications are the most apparent symptoms. Guillain-Barre syndrome (GBS) is an acute immune-mediated polyradiculoneuropathy often related to previous infectious exposure. GBS emerged as a potentially severe complication of coronavirus disease 2019 (COVID-19) since its declaration as a global pandemic. We report the first case of COVID-19-induced acute motor axonal neuropathy variant of Guillain-Barre syndrome (GBS) from New York, USA. Our patient was a 66-year-old woman who had recently tested positive for COVID-19 and presented with bilateral upper and lower extremity weakness. Electromyogram studies showed acute demyelinating polyradiculoneuropathy. She was diagnosed with an acute motor axonal neuropathy variant of GBS. She was successfully treated with intravenous immunoglobulins (IVIGs) with marked improvement. In six months, she regained her strength back to normal. Whether GBS incidence in COVID-positive patients is based on molecular mimicry or anti-ganglioside antibodies is unclear. Physicians should be aware of GBS as a potentially serious complication associated with COVID-19. Further investigations and trials should be conducted better to understand the mechanism of GBS in patients of COVID-19.

## Introduction

On December 31, 2019, a novel coronavirus (COVID-19) was detected in Wuhan City, Hubei Province of the People's Republic of China [1]. COVID-19 is a new beta coronavirus, which acts via fusion with the angiotensin-converting enzyme 2 (ACE2) receptor [2]. COVID-19 viruses are emerging as a multisystem and autoimmune disease [3]. The first series of patients reported in December 2019 were typically diagnosed with a pneumonia-like presentation. The most common clinical symptoms were fever, cough, dyspnea, myalgia, headache, and diarrhea [4]. It can invariably affect the multisystem organs and trigger the autoimmune phenomenon [3,4]. Several neurological manifestations have been associated with this disease. These manifestations involved both the central and peripheral nervous systems [5].

There are emerging cases of COVID-19-induced neuropathy and Guillain-Barre syndrome (GBS) reported worldwide [6]. That correlation can be considered a direct effect of the virus on the nervous system or post-infectious immune-mediated response. GBS is acute immune-mediated polyneuropathy with several variant forms. GBS is marked by ascending motor impairment, mild to severe sensory disturbances, cranial nerve involvement, autonomic symptoms, and muscle or radicular pain. It is thought to be due to the molecular mimicry phenomenon, usually preceding infection best understood with Campylobacter jejuni related GBS. The most associated organisms are Campylobacter jejuni, cytomegalovirus, Epstein-Barr virus, human immunodeficiency virus (HIV), and Zika virus. This report describes GBS symptoms in one infected patient with COVID-19, seen for the first time in New York, USA.

## Case presentation

A 66-year-old Hispanic woman with a past medical history of hypertension presented to the Emergency Department (ED) with a one-week history of shortness of breath and cough. On initial examination, she had worsening hypoxia to 50s and confusion. Prior to the presentation, she had tested positive for COVID-19 with reverse-transcription polymerase chain reaction (RT-PCR) test at an outside clinic one week ago. She denied fever, chest pain, nausea, vomiting, diarrhea, fatigue, myalgia, arthralgia, loss of consciousness, and neurological deficits. She was transferred to the intensive care unit (ICU) due to hypoxic respiratory failure requiring noninvasive positive pressure ventilation (NIPPV) and hemodynamic instability.

**Figure 1:**
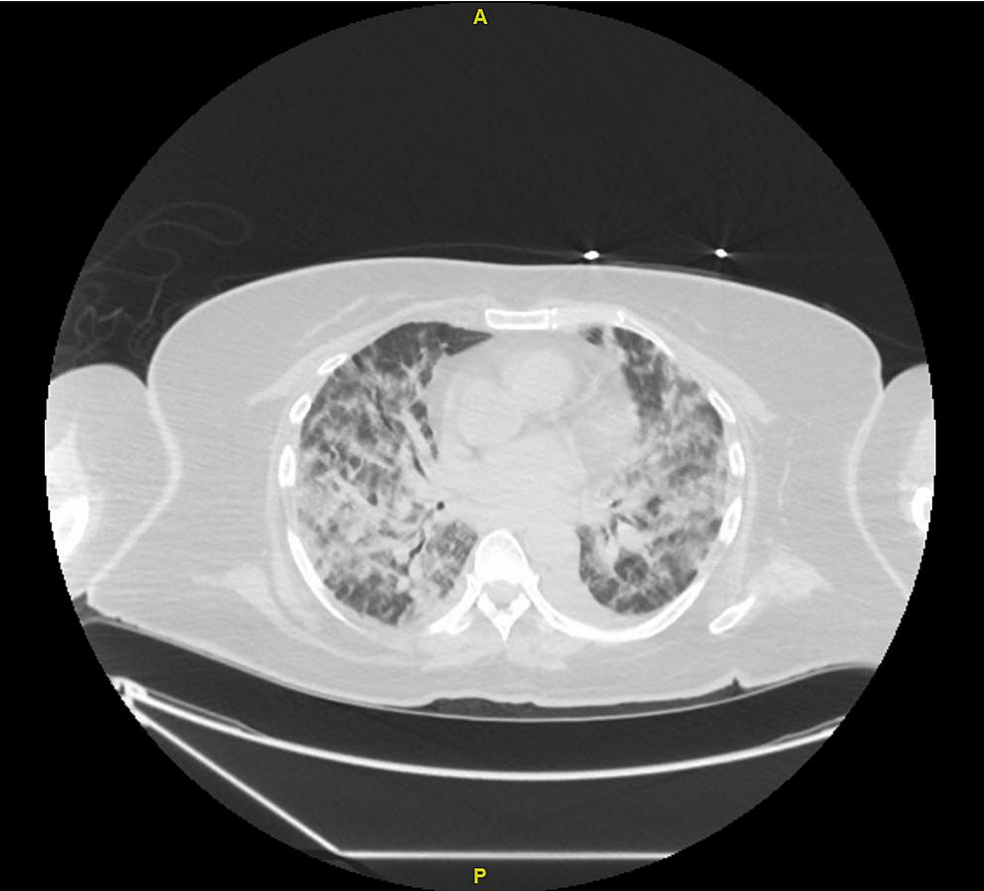
Computed tomography (CT) of chest showing bilateral diffuse patchy infiltrates compatible with COVID-19 pneumonia

While in the ICU, she was conscious, alert, and oriented with normal speech and higher mental functions on the neurological exam. On chest examination, she had bilateral rhonchi with decreased air entry. She denied the use of cigarettes, alcohol, or other recreational drugs. Later in the ICU, she developed severe respiratory distress and worsening hypoxia despite maximum oxygen support and had to be intubated. Her chest X-ray and chest computed tomography (CT) show bilateral diffuse patchy infiltrates compatible with COVID-19 pneumonia, as shown in Figure 1. She had elevated inflammatory markers, including white blood cells, C-reactive protein, lactate dehydrogenase, ferritin, and D-dimer. Her initial laboratory investigations are mentioned in Table 1. Her COVID-19 was treated with dexamethasone 6 mg twice a day for 10 days and broad-spectrum antibiotics to cover bacterial pathogens. Remdesivir and baricitinib were not given due to transaminitis present at admission.

**Table 1:** Initial laboratory investigation

Parameter	Value	Normal Range
RBC Count	3.5	[4.00 - 5.20 MIL/ul]
Hemoglobin	10.5	[12.0 - 16.0 g/dl]
Hematocrit, Whole Blood	32.2	[42.0 - 51.0%]
Platelet	314	[150 - 400 k/ul]
White Blood Cell Count	15.7	[4.8 - 10.8 k/ul]
Neutrophil %	88.4	[40.0 - 70.0%]
Lymphocyte %	5.5	[20.0 - 50.0%]
Sodium, Serum	140	[135 - 145 mEq/L]
Potassium, Serum	4.8	[3.5 - 5.0 mEq/L]
Chloride, Serum	101	[98 - 108 mEq/L]
Bicarbonate, Serum	30	[24 - 30 mEq/L]
Blood Urea Nitrogen, Serum	37	[6 - 20 mg/dL]
Creatinine, Serum	0.8	[0.5 - 1.5 mg/dL]
Calcium, Total Serum	8.6	[8.5 - 10.5 mg/dL]
Magnesium, Serum	3.1	[1.5 - 2.7 mg/dL
Phosphorous	2.1	[2.5 - 4.5 mg/dL]
Total Protein Serum	5.9	[5.8 - 8.3 g/dl]
Albumin, Serum	2.7	[3.2 - 4.6 g/dl]
Alanine Aminotransferase, Serum	361	[5 - 40 unit/L]
Aspartate Transaminase, Serum	192	[9 - 36 unit/L]
Alkaline Phosphatase, Serum	100	[43 - 160 unit/L]
Bilirubin, Serum Total	0.2	[0.2 - 1.1 mg/dL]
Bilirubin, Serum Direct - Conjugated	0.1	[0.0 - 0.3 mg/dL]
Cholesterol, Serum	105	[170 - 240 mg/dL]
Low Density Lipoprotein (LDL)	53	[<=160 mg/dL]
High Density Lipoprotein Cholesterol, Serum	28	[34 - 82 mg/dL]
Triglycerides, Serum	119	[60 - 150 mg/dL]
Thyroid Stimulating Hormone, Serum	0.17	[0.40 - 4.50 mIU/L]
Lactate Dehydrogenase (LDH)	602	[110 - 210 unit/L]
C-Reactive Protein, Serum	301.6	[<=5.00 mg/L]
D-Dimer Assay, Plasma	2821	[0 - 230 ng/mL]
Ferritin	957	[13.0-150.0 ng/mL]
Pro Brain Natriuretic Peptide (BNP)	3656	[0 - 125 pg/mL]
Creatine Kinase, Serum	33	[20 - 200 unit/L]

The hospital course was complicated by septic shock requiring multiple vasopressors, vaginal bleeding, and shock liver with multi-drug resistant (MDR) Acinetobacter and Pseudomonas pneumonia and persistently elevated D-dimer. She eventually developed acute respiratory distress syndrome (ARDS) and was placed on low positive end-expiratory pressure (PEEP) and high FiO2 as per the ARDS protocol. She failed multiple weaning trials from the ventilator and underwent a tracheostomy and percutaneous endoscopic gastrostomy (PEG) placement.

**Figure 2:**
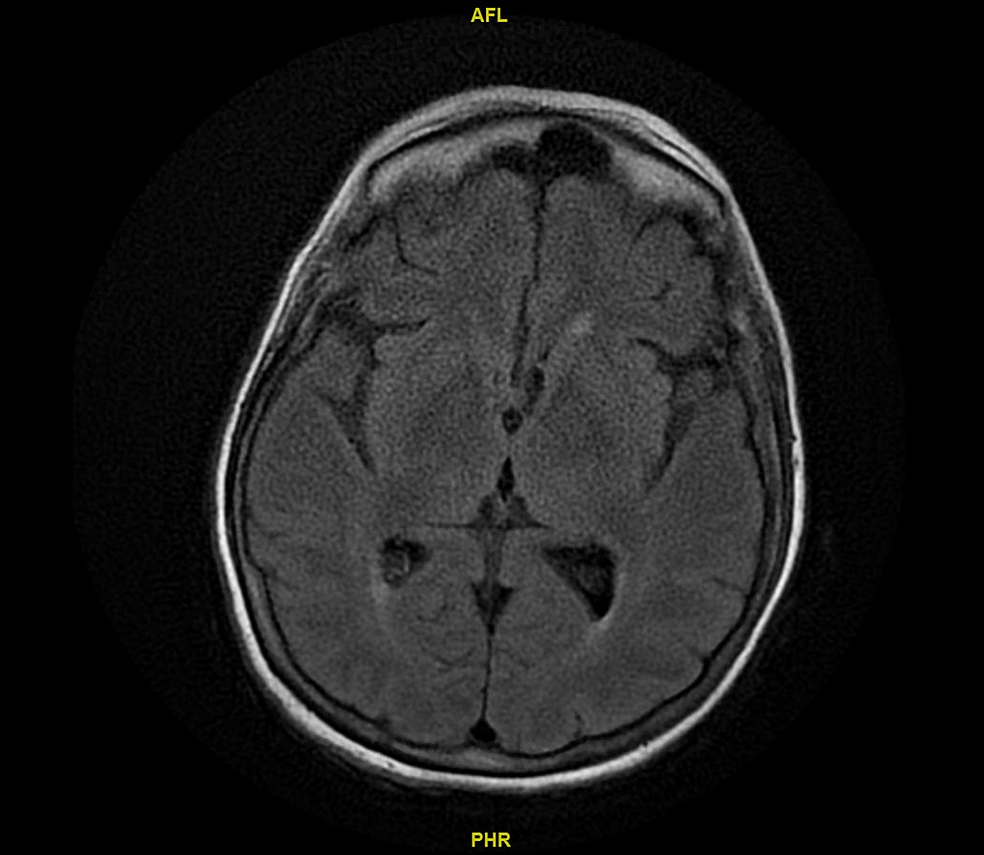
MRI head showing normal architecture

Later in the hospital course, she developed bilateral upper and lower extremity weakness, predominantly upper extremities. On exam, power in bilateral upper extremities was 0/5, right lower extremity was 3/5, and left lower extremity was 2/5. The sensory sensation was intact in all four extremities. The patient was suspected of having critical illness myopathy. Computed tomography (CT) scan and magnetic resonance imaging (MRI) of the head showed no evidence of acute intracranial hemorrhage, mass effect, midline shift, or recent infarct, as shown in Figure 2. CT cervical spine showed degenerative spondylosis with moderate to severe central canal stenosis from the C4/5 through C6/7 levels. Neurology was consulted, and electromyography (EMG) was requested. Family members declined lumbar puncture and cerebrospinal fluid (CSF) analysis. Anti-GQ1b ganglioside antibody was negative at <1:100.

**Figure 3:**
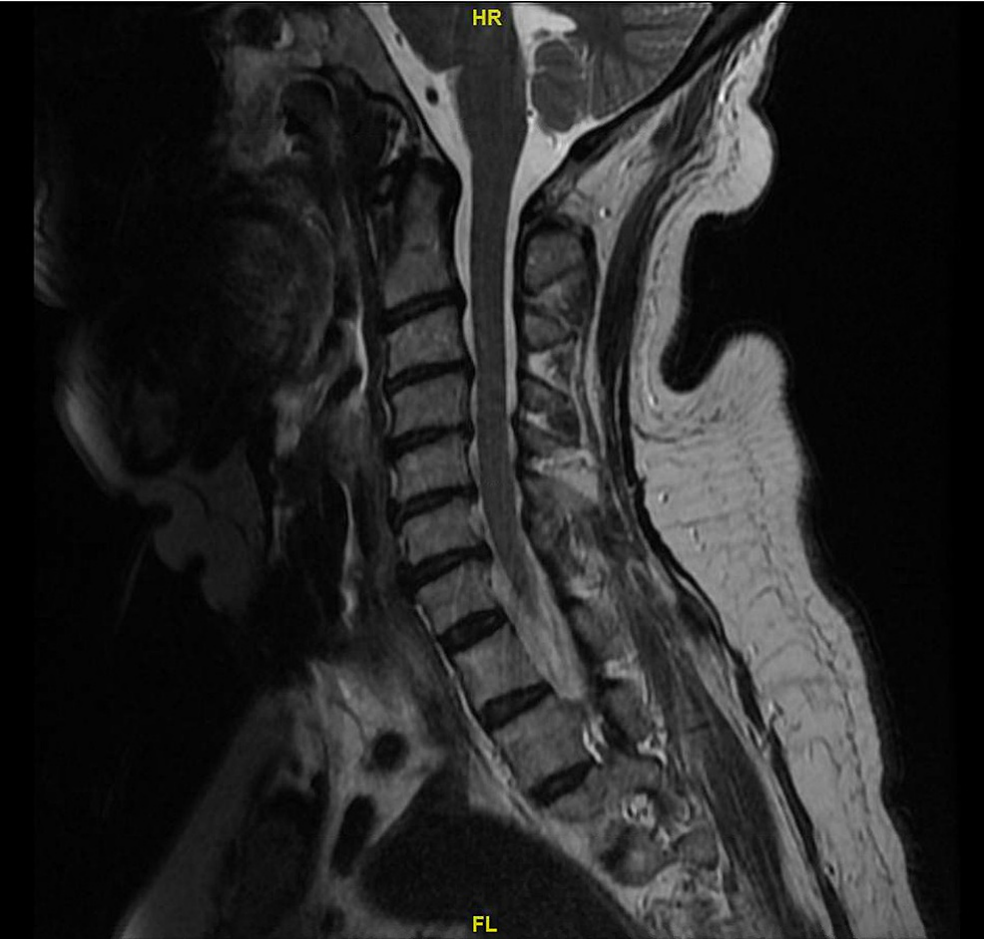
MRI cervical spine showing cervical radiculopathy

The electrodiagnostic study revealed sensorimotor axonal multifocal peripheral neuropathy predominantly affecting upper extremities, as shown in Tables 2-4. EMG reported reduced amplitude in the right median, ulnar, and peroneal motor compound muscle action potential (CMAP) with normal distal latencies and conduction velocities, absent right radial and medial sensory nerve action potentials (SNAP), normal F-wave latency in the right ulnar, peroneal and tibial. There was likely some degree of cervical radiculopathy on MRI, as shown in Figure 3. However, the absent sensory responses pointed to a component of neuropathy. There also appeared to be sural sparing, which can be seen in Guillain-Barré syndrome (GBS), as shown in Tables 2-4. She was diagnosed with an axonal variant of GBS. The patient was started on aggressive physiotherapy and intravenous immunoglobulin (IVIG). She received 0.4 g/kg/day (2gm in total) of intravenous immunoglobulins (IVIG) for five days, and she had marked improvement of her symptoms. Motor and sensory examination post-IVIG revealed bilateral upper extremities' power improving to 2/5, with the movement of fingers. Improving muscle power to lower extremities (LE), left lower extremity (LLE): 4/5, right lower extremity (RLE): 3/5, the sensation was intact in all four extremities.

**Table 2:** Electrodiagnostic results

Side	Muscle	Nerve	Root	Ins Act	Fibs	Psw	Amp	Dur	Poly	Recrt	Int Pat
Right	Abd Poll Brev	Median	C8-T1	Incr	2+	2+	Nml	Nml	0	Nml	Decr
Right	1^st^ Dor Int	Ulnar	C8-T1	Incr	3+	3+	Nml	Nml	0	Nml	Decr
Right	Ext Dig Com	Radial (Post Int)	C7-8	Incr	2+	2+	Nml	Nml	0	Nml	Decr
Right	Biceps	Musculocut	C5-6	Incr	2+	2+	Nml	Nml	0	Nml	Decr
Right	Ant Tibialis	Dp Br Peron	L4-5	Nml	Nml	Nml	Nml	Nml	0	Nml	Decr
Right	Med Gastroc	Tibial	S1-2	Nml	Nml	Nml	Nml	Nml	0	Nml	Decr
Right	Vastus Lat	Femoral	L2-4	Nml	Nml	Nml	Nml	Nml	0	Nml	Decr
Right	Vastus Med	Femoral	L2-4	Nml	Nml	Nml	Nml	Nml	0	Nml	Decr
Right	Gracils	Obturator	L2-4	Nml	Nml	Nml	Nml	Nml	0	Nml	Decr

**Table 3:** Electromyography (EMG) motor nerves

Site	Onset (ms)	Norm Onset (ms)	O-P Amp (mV)	Norm Amp (mV)	Neg Dur (ms)	Segment Name	Delta-O (ms)	Dist (cm)	Vel (m/s)	Norm Vel (m/s)
Right Median (Abd Poll Brev)										
Wrist	3.75	<4.2	0.47	>4.0	9.38	Elbow-Wrist	4.53	22	48.57	>49
Elbow	8.28		0.36		9.06					
Right Ulnar (Abd Dig Min)										
Wrist	2.89	<3.3	0.87	>6.0	6.48	B Fib-Ankle	5.55	26	46.85	>40.0
Right Peroneal (EDB)										
Ankle	4.92	<6.5	1.13	>2.0	6.80					
B Fib	10.447		1.05		6.72					
Right Tibial (AHB)										
Ankle	5.39	<6.7	5.16	>4.0	5.93	Knee-Ankle	7.50	35	46.67	>40.0
Knee	12.89		4.34		5.78					

**Table 4:** Electromyography (EMG) sensory nerves

Site	NR	Peak (ms)	Norm Peak (ms)	O-P AMP (μV)	Norm Amp (μV)	Segment Name	Delta-O (ms)	Dist (cm)	Vel (m/s)	Norm Vel (m/s)
Right Median Anti (2^nd ^Digit)										
Wrist	NR		<3.5		>20.0					
Right Ulnar Anti (5^th^ Digit)										
Wrist		2.72	<3.1	27.8	>10.0	Wrist-5^th^ Digit	2.09	11	52.63	>50.0
Right Radial Anti (Base 1^st^ Dig)										
Wrist	NR		<2.7							
Right Sural (Lateral Mall)										
14 cm		2.53	<4.4	42.43	>6.0	14cm-Lat Mall	1.94	9	46.39	>40.0

The patient was discharged to a nursing home for rehabilitation purposes. Three months after the post-discharge patient was successfully liberated from the ventilator and was placed on home oxygen. The patient's neurological signs and symptoms improved significantly over time. The patient did not have any short or long-term treatment complications from intravenous immunoglobulins.

## Discussion

Sars-CoV-2 is one of the types of coronaviruses that causes severe lung infection. While the primary presentation of COVID-19 is respiratory, neurological manifestations and complications are increasing [5]. Guillain-Barre syndrome (GBS) is emerging as a relevant disease that may appear in COVID-19 patients. Sars-CoV-2 mechanism of action is believed to be accessing angiotensin-converting enzyme 2 (ACE2) receptors [2]. ACE2 receptors are present in large numbers in the lung, gastrointestinal tract, cardiomyocytes, urothelial cells, and proximal tubular cells, affecting these systems. The nervous systems, including neurons and glial cells, also express ACE2 receptors, making it a potential target of Sars-CoV-2 [7].

The first reported case of COVID-induced GBS was from Wuhan, China, which refers to a woman who presented with acute lower extremity weakness and areflexia after four days of returning to Shanghai from Wuhan progressed over three days to the arms without any systemic symptoms. Italy has also reported six patients with GBS [3]. All presented with acute onset of upper and lower extremity weakness, distal paresthesia's, and sensory deficits 3-10 days after experiencing cough, anosmia, ageusia, and sore throat [8]. GBS is an immune-mediated disorder; molecular mimicry and production of anti-ganglioside antibodies are considered one of the mechanisms of autoimmune disorder that plays an important role [9]. The mechanism of GBS formation in patients infected with COVID-19 has not yet been investigated. Studies on coronaviruses have been shown that neurophilic and neuroinvasive characteristics are present [10]. Although no studies have been directly performed about COVID-19, the arrangement of SARS is very similar to that of COVID-19. One hypothesis shines a light on COVID-19 stimulation of inflammatory cells and the production of various inflammatory cytokines, resulting in the initiation of immune-mediated processes [11]. Our patient had anti-GQ1b ganglioside antibody negative. The rest of the ganglioside panel was not available. Before the COVID-19 pandemic, it was rare to have a negative ganglioside antibody panel for GBS. There was only one case report with GM1 ganglioside antibody and COVID-19-related GBS [12]. Our case adds to the previously published literature review showing most COVID-19-induced GBS were ganglioside antibody negative pointing towards a different target protein [12].

GBS presents as progressive ascending weakness of limbs and reduced or loss of deep tendon reflexes. The protein concentration is markedly increased with normal white blood cells on CSF analysis. GBS usually precedes a virus or bacterial infection. The symptoms usually peak within four weeks. Our patient was also noted to develop bilateral upper and lower extremities weakness almost two weeks post-COVID diagnosis. Electromyography (EMG) was also suggestive of GBS. The patient's weakness improved after receiving intravenous immune globulin (IVIG). The axonal variant of GBS takes time to recover due to the nature of the injury but in this case, due to early and prompt IVIG treatment the patient recovered quickly. Months later, the patient was liberated from the ventilator and continued home oxygen. It is crucial to follow long-term sequelae to assess the extent of improvement after receiving intravenous immune globulin. Further investigations and trials should be conducted to better understand the mechanism of GBS in patients of COVID-19. Regarding GBS, clinicians should keep a high suspicion of patients who present with paresthesia and trouble moving after COVID-19 infection [13].

## Conclusions

Physicians should be aware of GBS as a rare complication associated with COVID-19. All patients with neurological deficits with sensory, motor, or autonomic symptoms should be screened for GBS. GBS is an emerging and relevant neurological disease in COVID-19 patients. Its pathophysiology and both clinical and electrophysiological characteristics remain to be further studied. Early diagnosis and management can improve clinical outcomes. Keeping in view the course of the presentation of GBS, therapy with IVIG should be considered along with antiviral treatment to prevent grave complications. It is yet to be confirmed if GBS in COVID patients is caused by an immune response due to molecular mimicry or the production of anti-ganglioside antibodies. More and more studies are needed to fully understand the nature of COVID-19-induced neuropathies and GBS and treatment outcomes. Longitudinal follow-ups are equally essential to comprehend long-term sequelae and outcomes fully.
